# Development of L-Lysine-Loaded PLGA Microparticles as a Controlled Release System for Angiogenesis Enhancement

**DOI:** 10.3390/pharmaceutics15020479

**Published:** 2023-02-01

**Authors:** Nunzia Gallo, Stefano Quarta, Marika Massaro, Maria Annunziata Carluccio, Amilcare Barca, Donato Cannoletta, Luisa Siculella, Luca Salvatore, Alessandro Sannino

**Affiliations:** 1 Department of Engineering for Innovation, University of Salento, Via Monteroni, 73100 Lecce, Italy; 2 Department of Biological and Environmental Sciences and Technologies (DISTEBA), University of Salento, 73100 Lecce, Italy; 3 Institute of Clinical Physiology (IFC), National Research Council (CNR), 73100 Lecce, Italy

**Keywords:** L-lysine, PLGA, drug delivery systems, microspheres, angiogenesis

## Abstract

Vascularization is a highly conserved and considerably complex and precise process that is finely driven by endogenous regulatory processes at the tissue and systemic levels. However, it can reveal itself to be slow and inadequate for tissue repair and regeneration consequent to severe lesions/damages. Several biomaterial-based strategies were developed to support and enhance vasculogenesis by supplying pro-angiogenic agents. Several approaches were adopted to develop effective drug delivery systems for the controlled release of a huge variety of compounds. In this work, a microparticulate system was chosen to be loaded with the essential amino acid L-lysine, a molecule that has recently gained interest due to its involvement in pro-angiogenic, pro-regenerative, and anti-inflammatory mechanisms. Poly (lactic-co-glycolic acid), the most widely used FDA-approved biodegradable synthetic polymer for the development of drug delivery systems, was chosen due to its versatility and ability to promote neovascularization and wound healing. This study dealt with the development and the effectiveness evaluation of a PLGA-based microparticulate system for the controlled release of L-lysine. Therefore, in order to maximize L-lysine encapsulation efficiency and tune its release kinetics, the microparticle synthesis protocol was optimized by varying some processing parameters. All developed formulations were characterized from a morphological and physicochemical point of view. The optimized formulation was further characterized via the evaluation of its preliminary biological efficacy in vitro. The cellular and molecular studies revealed that the L-lysine-loaded PLGA microparticles were non-toxic, biocompatible, and supported cell proliferation and angiogenesis well by stimulating the expression of pro-angiogenic genes such as metalloproteinase-9, focal adhesion kinases, and different growth factors. Thus, this work showed the potential of delivering L-lysine encapsulated in PLGA microparticles as a cost-effective promoter system for angiogenesis enhancement and rapid healing.

## 1. Introduction

Vascularization plays a key role in tissue homeostasis, repair, and healing because it is essential to the adequate exchange of nutrients, waste, gases, and signals. New vessels in the body are formed via three processes: vasculogenesis, angiogenesis, and arteriogenesis [1]. Vasculogenesis occurs in the early stages of the embryonic development and gives rise to the primitive circulatory system. Angiogenesis and arteriogenesis occur in adult tissues. In particular, angiogenesis consists of the sprouting, growth, and maturation of new vessels from existing vasculature in response to tissue hypoxia or insufficient tissue oxygen tension, while arteriogenesis occurs in cases of arterial occlusion and consists of the remodeling of pre-existing vasculature in functional arteries [1]. In both processes, endothelial cells play a key role in orchestrating the entire process by undergoing profound morphological, metabolic, and functional modulations [2]. Angiogenesis is a sophisticated multistep process that is finely regulated by the interaction of the extracellular matrix components with growth factors and inhibitors, an imbalance in which leads to process dysregulation and thus to vascular system diseases. Insufficient angiogenesis is responsible for blood vessels’ insufficient growth, which leads to poor blood flow and tissue death, thereby requiring external intervention to support them [3]. Thus, although vascularization is a complex and finely regulated process, it can be slow or inadequate in cases of hard-to-heal wounds.

As a result, several strategies based on pro-angiogenic agents were developed to support or enhance vasculogenesis. However, there are some issues that are related to the administration of bioactive agents such as their fast clearance, dose-dependent side effects, and therapeutic concentration maintenance during time. In this circumstance, tissue engineering and regenerative medicine moved forward the development of biomaterial-based strategies to enhance vasculogenesis by providing pro-angiogenic agents in a controlled manner over time. In particular, several nano- and microparticle-based drug delivery systems (DDSs) were developed for the controlled release of a huge variety of compounds such as growth factors (i.e., vascular endothelial, platelet-derived, and placental growth factors), cells (i.e., human mesenchymal stem cells and Chinese hamster ovary cells), cell-derived materials (i.e., exposomes derived from adipose stem cells), decellularized matrices (i.e., the human placental matrix), antibodies (i.e., bevacizumab), peptides, DNA (i.e., polydeoxyribonucleotide), and RNA [1,4,5,6,7,8,9,10,11,12,13,14].

In this work, with the ultimate purpose of developing advanced therapies for the treatment of hard-to-heal wounds, a new drug-loaded, microparticulate-based delivery system was developed in order to support angiogenesis and regenerative processes. Aside from the compounds listed above, the essential amino acid L-lysine (LYS or K), a molecule that has recently gained interest due to its many advantages, was chosen as the bioactive agent to be encapsulated in a microparticulate system. LYS is physiologically implicated in a series of biological functions such as collagen synthesis; tissue growth and repair; and the production of antibodies, hormones, and enzymes [15]. Several works reported the ability of LYS to mediate angiogenesis in wound healing [16,17,18,19,20]. Indeed, in a randomized, open-label, prospective study, an LYS-based cream (15%) was able to significantly improve the healing rate and quality of the wound from the second week onward with less scarring and pain in cases of second-degree superficial burns compared to the control group (69% in the LYS-treated group and 42% in the control group) [19]. Another study demonstrated that an LYS-based cream (15%) also was effective in cases of non-diabetic foot ulcers [20]. The angiogenic property of LYS also was demonstrated in cases of ischemia. The intravenous administration of LYS (60 mg/kg) in patients with symptoms of acute hemispheric stroke resulted in a marked improvement of its regression in terms of cerebral blood flow and volume as well as in mean transit time ratios, which was probably due to LYS-induced angiogenesis [21]. It was hypothesized that LYS allows reparative cells and serum growth factors to enter into the wound beds and augment the healing process in situ [19]. From a mechanistic point of view, LYS acts as a cell surface bridge and enhances the binding force between angiogenic factors and the corresponding receptors, thereby leading to an enhanced biological response [16,18]. The effect of LYS could also be due to the fact that the basic amino acids, which include arginine, histidine, and LYS, are naturally “vasoactive” in addition to their usual metabolic functions [18]. Moreover, it should be considered that because it belongs to the class of essential amino acids, LYS is not recognized as a foreign substance but as a molecule whose catabolism and anabolism physiologically occurs using the existing cellular metabolism enzymes [22]. Indeed, no local or systemic side effects have ever been observed in clinical studies [19,20,21,22]. Thus, LYS was proposed as an alternative therapeutic agent to be administered in a controlled manner over time due to some advantages in its use such as its pro-angiogenic, pro-healing, and anti-inflammatory activity as well as its low cost, easy harvesting, high biocompatibility, complete biodegradability, and lack of local or systemic adverse effects.

To prepare particles with controlled properties such as size, release kinetics, and degradation rates, it is important to choose not only the right method but also the right materials and synthesis parameters (i.e., type and concentration of surfactant, solvent and polymer, active agent properties, and the type and extent of energy input of sonication or homogenization) and to finely control them. The commonly used biomaterials for the manufacture of DDSs are from both natural and synthetic sources. Among them, poly (lactic-co-glycolic acid) (PLGA), a U.S. Food and Drug Administration (FDA)-approved co-polymer of lactic and glycolic acid, is one of the most widely used synthetic biomaterials for the manufacture of several types of implantable devices (e.g., nano- or microparticles, implants, nanogels, nanofibers, and thin films) [23,24,25,26]. PLGA is a highly versatile, biocompatible, almost atoxic, and good manufacturing practice (GPM)-available polymer. The possibility to tune PLGA’s properties in terms of the degradation time by altering its monomer ratio and its molecular weight allows the production of different types of PLGA that have different hydrolysis rates that can vary from weeks to years and thus can be customized according to the need. Moreover, its monomer nature makes it and its byproducts highly biocompatible, bioactive, and almost completely free of toxicity [4]. PLGA has been shown to be an effective material for protecting and modulating the sustained release of many types of drugs [23,27,28]. For all these reasons, many PLGA-based DDSs have been approved and are commercially available worldwide; e.g., Risperdal Consta^®^, Zilretta^®^, Vivitrol^®^, Signifor^®^ LAR, Lupaneta Pack^TM^, Nutropin Depot^®^, and others [23,27]. Moreover, a few studies reported how PLGA alone could exert a therapeutic effect that was triggered by its hydrolysis products [5,29,30]. Its bioactivity is due to lactic acid metabolism, which spurs the expression of the pro-angiogenic factors interleukin 8 (IL-8), vascular endothelial growth factor (VEGF), and VEGF receptor 2 (VEGFR2) as well as collagen deposition. It also downregulates the expression of inflammatory cytokines produced by macrophages [4].

In this work, we decided to test the pro-angiogenic efficacy of a PLGA-based microparticle system (MS) loaded with LYS. The preparation method chosen for the development of the LYS-loaded PLGA MSs was [31,32,33] the double-emulsion solvent-evaporation technique [31]. This is a multistep procedure that is usually employed for hydrophilic drug encapsulation. Basically, an aqueous solution containing the drug is added to an organic phase that consists of the polymer (i.e., PLGA) and an organic solvent (e.g., dichloromethane, ethyl acetate, dimethyl formamide, and so on) by means of vigorous stirring to form the first water-in-oil (*w*/*o*) emulsion [23,31]. The first emulsion is then dispersed into another aqueous phase that contains a surfactant (e.g., PVA) to form the second water-in-oil-in-water (*w*/*o*/*w*) emulsion [34]. Then, solvent extraction and evaporation are conducted in a process that allows the PLGA molecules to undergo coalescence, which leads to the formation of microparticles with an inner honeycomb structure [23,35]. In this step, part of the drug can migrate to the external aqueous phase [35,36]. After the solvent is completely extracted from the microparticles, the hardened microparticles are collected via centrifugation and dried via freeze-drying [23,31,34]. The lyophilized form of the microparticles can be stored for several years [31]. One of the advantages of emulsification methods is that they can be easily scaled up [32,33].

To the best of our knowledge, this was the first study that dealt with the development, optimization, characterization, and preliminarily effectiveness evaluation of a microparticulate system for the controlled release of LYS. Due to each drug’s unique physicochemical properties, each formulation requires an ideal processing-condition combination for ad hoc microparticle system development [23]. The interactions among the drug, the polymer, the solvent, and the temperature unpredictably affect microparticle properties in a non-linear way [23,34,37,38]. Thus, in order to maximize the LYS encapsulation efficiency, and MS synthesis protocol was optimized by varying some of the processing parameters. All of the developed formulations were characterized from morphological and physicochemical points of view using scanning electron microscopy (SEM) and laser light diffraction. The MS yield, drug encapsulation efficiency, and release kinetics were assessed gravimetrically and by means of a colorimetric assay, respectively. The MS degradation was followed by SEM. Finally, the optimized formulation was biologically tested for its pro-angiogenic efficacy in a human endothelial cell model in vitro.

## 2. Materials and Methods

### 2.1. Materials

The PLGA (D,L-lactide/glycolide: 50/50; molecular weight: 30–60 kDa), polyvinyl alcohol (PVA), dichloromethane (DCM), and LYS were all of analytical grade and purchased from Sigma-Aldrich (Milan, Italy). The distilled water was obtained from the Merck KGaA Millipore Milli-U10 water purification facility (Darmstadt, Germany). If not otherwise stated, all other chemicals used were of analytical grade and purchased from Sigma-Aldrich (Milan, Italy).

### 2.2. MS Synthesis

**Figure 1:**
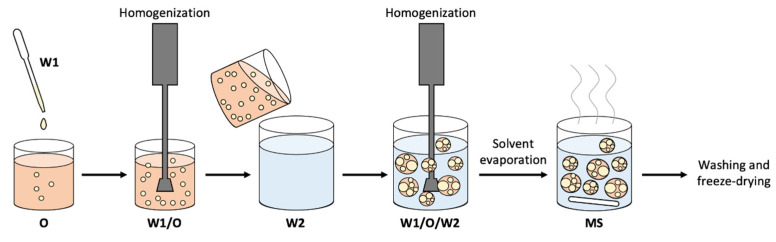
Exemplary scheme of the double-emulsion technique (W1/O/W2) used for the microsphere development.

The delivery system for the controlled release of LYS was developed by applying a double-emulsion solvent-evaporation method (Figure 1) [39]. The first water phase (W1) was prepared by dissolving LYS in 0.5 mL of distilled water. Subsequently, the W1 was added to 5 mL of oil phase (O) composed of 15% PLGA dissolved in DCM. The W1/O single emulsion was emulsified at 15,000 rpm for 2 min at 4 °C by means of an IKA T25 Digital Ultra Turrax overhead blender (IKA^®^-Werke GmbH & Co., KG, Breisgau, Germany). Next, the W1/O emulsion was added the second water phase (W2) of a 1% PVA and homogenized for 1 min at 8000 rpm to obtain the W1/O/W2 double emulsion. The DCM was allowed to evaporate at room temperature for 2 h at 4 °C under stirring in a flow hood. The W1, O, and W2 phases were always freshly prepared and cooled down to 4 °C before use. A low working temperature was chosen because it prevented protein from moving toward the surface during solidification, which left less protein near the surface and reached higher encapsulation efficiency values [36].

The so-obtained LYS-loaded MSs (MS/Ks) were then collected via centrifugation at 4000 rpm for 5 min, washed thrice with distilled water, and freeze-dried by means of a LIO-5P freeze-drier (Cinquepascal s.r.l., Trezzano sul Naviglio, Italy).

In order to maximize the encapsulation efficiency of the selected drug delivery system, some processing parameters were varied (Table 1). For each experimental condition, empty MSs also were produced.

### 2.3. Yield

The process effectiveness was evaluated by determining the yield of the produced MS/Ks, which was calculated as the percentage of the ratio between the weight of the freeze-dried MSs and the amount of the bioactive agent (LYS) and the polymer (PLGA) used in their preparation [28,40]. Thus, yields were calculated using the following formula:

(1) $$Yield\%=\frac{W_{dry\;MS}}{W_{PLGA}+W_{K}}\times 100$$

The test was repeated three times for each sample type. Statistical significance was assessed by mean of the Student’s *t*-test.

### 2.4. Morphological Analysis

The morphologies of the empty MSs and MS/Ks were examined using an SEM EVO^®^ 40 (Carl Zeiss AG, Jena, Germany). Approximately 1–2 mg of lyophilized empty MSs and MS/Ks were deposited onto a carbon adhesive disk mounted on an aluminum stub and directly observed at an accelerating voltage of 20 kV.

### 2.5. Diameters Distribution

The MSs’ diameter distribution was measured via laser light diffraction by means of a CILAS 1190L (CILAS, Orléans, France). About 20 mg of lyophilized empty MSs and MS/K were resuspended in distilled water, sonicated at 4 °C for 15 min, and analyzed to determine their diameter distribution. The assessment of the particle size distribution was conducted when the laser obscurations were between 2% and 4% [35]. The average particle size was expressed as the volume mean diameter (D_M_). The distribution width was described by the Span index, which was calculated using the following equation [39,41]:

(2) $$Span=\frac{D_{90}-D_{10}}{D_{50}}$$

where *D*_90_, *D*_50_, and *D*_10_ are the diameters for which 90%, 50%, and 10% of the particles were respectively below each value [39]. The test was repeated three times for each sample type. The statistical significance was assessed by means of a Student’s *t*-test.

### 2.6. Encapsulation Efficiency

To evaluate the LYS encapsulation efficiency, about 20 mg of lyophilized MS/K was hydrolyzed in 1 mL of NaOH 0.2 M and SDS 5% for 24 h at room temperature [33]. The aqueous extract was collected as the supernatant after centrifugation at 4000 rpm for 5 min at room temperature. The LYS quantity present in the extract was spectrophotometrically determined by means of a slightly modified TNBS test [42,43]. About 0.2 mL of extract was added to 0.3 mL of 4% (*w*/*v*) NaHCO_3_ solution and 0.5 mL of 0.05% (*w*/*v*) TNBS. Then samples were hermetically sealed and heated at 40 °C for 2 h [43]. After that, 1.5 mL of 6 M HCl was added before heating the samples at 60 °C for 90 min [42,43]. The reaction mixture was thus was brought to a final volume of 5 mL with distilled water and cooled down to room temperature. The sample absorbance at 320 nm was acquired using a UV spectrophotometer. Control samples were prepared using the same procedure except that HCl was added before adding the TNBS solution in order to inhibit its reaction with LYS [44]. The control sample absorbance was subtracted from each sample type’s absorbance. The eMSs were also analyzed in order to assess and remove unspecific reaction of the TNBS assay with MSs or MS residues. The calibration line was obtained from the correlation of the absorbance with 0.1 mg/mL of LYS in 4% (*w*/*v*) NaHCO_3_ with several concentration dilutions. The percentage of LYS encapsulation efficiency (*EE*%) was obtained as the ratio between the effectively encapsulated LYS (*LYS_f_*) and the LYS added in the primary emulsion (*LYS_i_*) as reported in Equation (3) [32,33,40]. The test was executed in triplicate for each sample type and statistically evaluated using a Student’s *t*-test.

(3) $$EE\%=\frac{LYS_{f}}{LYS_{i}}\times 100$$

### 2.7. Release Kinetics

About 40 mg of lyophilized MS/Ks was resuspended in 1.5 mL 0.01 M PBS (pH 7.4) and kept at 37 °C under continuous shaking for 3 days [40,45]. At fixed time points, samples were centrifuged (10,000 rpm for 1 min), and 1.0 mL of supernatant was removed before replacing it with fresh 0.01 M PBS (pH 7.4). The supernatant was then analyzed by means of a TNBS test as described in Section 2.6. The LYS release profile from the MSs was obtained by plotting the cumulative release versus the release time as determined using the equation below [46]:

(4) $$Cumulative\;release=C_{n}\times V_{t}+V_{s}\sum C_{n-1}$$

where *C_n_* is the concentration of LYS in the release medium at time *t*, *V_t_* is the release medium volume, and *V_s_* is the release medium volume withdrawn and subjected to TNBS testing. For the empty MSs, we followed the same procedure for the MS/K samples in order to remove the unspecific absorbance of the empty MSs from the LYS absorbance. The test was repeated 6 times for each sample type. The statistical significance was assessed by means of a Student’s *t*-test.

### 2.8. Degradation Resistance

The MS and MS/K degradation resistance was only qualitatively observed via SEM. About 10 mg of lyophilized MS/Ks was resuspended in 1.5 mL 0.01 M PBS, 0.01% of sodium azide, and 0.005% of PVA (pH 7.4) and kept at 37 °C under continuous shaking for 30 days. At fixed time points, samples were freeze-dried and approximately 1–2 mg of lyophilized empty MSs and MS/Ks was sprinkled onto a carbon adhesive disk mounted on an aluminum stub and observed at an accelerating voltage of 20 kV using a SEM EVO^®^ 40 (Carl Zeiss AG, Germany).

### 2.9. In Vitro Biological Assays

#### 2.9.1. Cell Culture and Treatments

The HMEC-1 was provided by E.W. Ades (Centers for Disease Control, Atlanta, USA) [47]. Cells were cultured in MCDB-131 medium supplemented with 15% FBS as previously described [48]. For cell treatments, the HMEC-1 (seeded at the density indicated in the legend figures) was cultured in MCDB-131 containing 15% FBS in the absence or presence of free LYS (90 and 180 µmol/L corresponding to the maximum cumulative release of LYS from MS/K-4 over 72 h), MS-4 (0.5 and 1.0 mg/mL), and MS/K-4 (0.5 and 1.0 mg/mL) for 24 to 72 h.

#### 2.9.2. Cell Viability

Cell viability was determined via the 3-(4,5-dimethylthiazol-2-yl)-2,5-diphenyltrazolium bromide (MTT) assay, a method that is based on the metabolic activity of viable cells to convert MTT into an insoluble formazan precipitate that can be quantified spectrophotometrically. In brief, after treatment with MSs, MS/Ks, or free LYS, cells were washed twice and then incubated with MTT (final concentration: 0.5 mg/mL) for 2 h, and the formazan products were dissolved with isopropanol. The absorbance was then measured at 595 nm using a microplate reader.

#### 2.9.3. Cell Migration Assay

The migration of HMEC-1 was assessed using a wound-healing assay [48]. The HMEC-1 was seeded in 6-well plates and grown to confluence. After reaching confluence, the monolayers were scratched with a sterile 200 μL pipette tip, washed twice, and incubated for 24 h in serum-depleted media in the presence or absence of free LYS (90 and 180 µmol/L), MS-4 (0.5 and 1 mg/mL), and MS/K-4 (0.5 and 1 mg/mL). After 24 h, cell migration into the denuded area was photographed under a phase-contrast microscope (10× objective) that was connected to a digital camera (EVOS XL Core Imaging System, Thermo Fisher Scientific, Waltham, MA, USA). The resulting images were analyzed using ImageJ software optimized with specific plugins for automatic detection of the scratched area [49]. The cell-empty area was measured, and treatments were compared. An increase in the cell-filled area indicated cell migration.

#### 2.9.4. RNA Isolation and Real-Time Quantitative Polymerase Chain Reaction

The total RNA was isolated using TRIzol reagent (Thermo Fisher Scientific, Waltham, MA, USA) according to the manufacturer’s protocol. RNA was retrotranscribed to cDNA as previously described [48]. Real-time PCR (qPCR) analyses were performed using the CFX96 Touch Real-Time PCR Detection System and software (Bio-Rad Laboratories, Segrate, Italy). All reactions were carried out in a total volume of 25 μL with 50 ng of cDNA, 0.3 pmol/L of the primer pair, and 12.5 μL 2× SYBR Green PCR master mix (Bio-Rad Laboratories, Segrate, Italy) under conditions that were previously described [48]. The quantifications were performed using the comparative critical threshold method (ΔΔCT), and the GAPDH and 18S genes were used as internal controls for normalization. The primer sequences used are listed in Table 2.

### 2.10. Statistical Analysis

All data were expressed as mean ± the standard deviation. The statistical significance of the experimental data was determined using a Student’s *t*-test. Differences were considered significant at *p* < 0.05.

## 3. Results

### 3.1. Yields

The effects of processing conditions on the yields are reported in Table 3. All processing conditions allowed us to recover a quantity of microspheres up to 70–80%. This result indicated that the double-emulsion technique, the chosen solvents, and the key process parameters (i.e., the temperature, ratio between the individual phase volumes, homogenization speed, and time) allowed us to transform almost all the raw polymer into microparticles. The highest yields were reached by increasing the PLGA and PVA concentrations up to 15% and 2%, respectively.

### 3.2. Morphological Analysis

**Figure 2:**
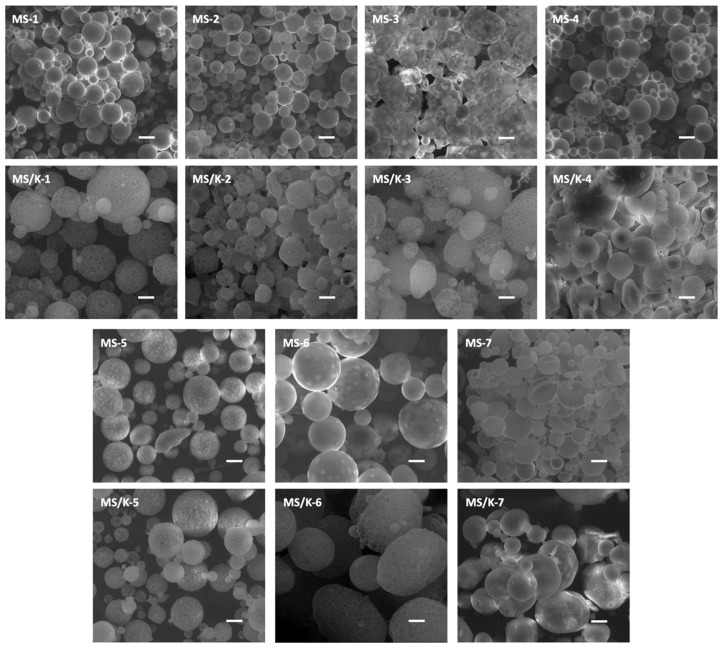
Representative SEM images of empty MSs and MS/Ks showing a spherical morphology. Scale bar: 20 μm. Magnification: 1000×.

SEM imaging confirmed the effective production of PLGA spherical structures of about 20–40 μm regardless of the processing conditions (Figure 2) [32,33]. However, processing conditions were found to deeply affect the microparticles’ superficial morphology. A smooth surface with small pores was observed for all empty formulations, which was in accordance with the literature [32]. In particular, the increase in the PLGA concentration led to a decrease in the surface pores, while the increase in the W2 volume led to an increase in the surface pores for the empty MSs. The addition of PBS in W2 seemed to not affect the empty particles’ surface morphology.

**Figure 3:**
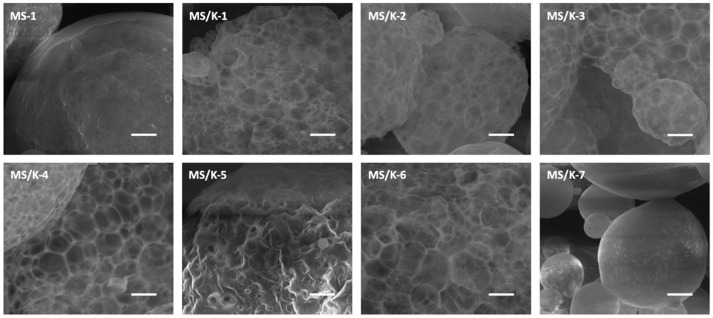
Effect of synthesis parameters on MS surface characteristics. Scale bar: 5 μm; magnification: 7000×.

**Figure 4:**
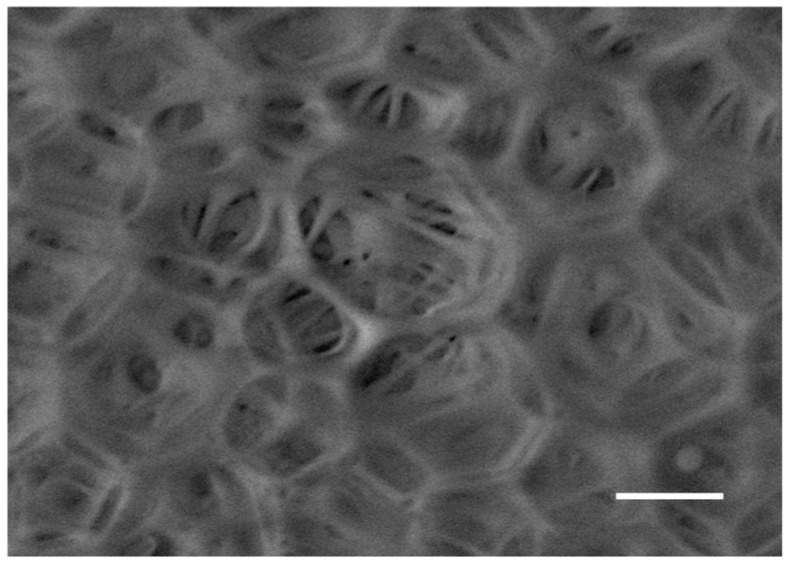
SEM micrograph of MS/K-4 wall structure. Scale bar: 3 μm; magnification: 13,000×.

LYS loading was found to deeply affect the particle morphology and showed the acquisition of a reticular structure in almost all cases (Figure 3). The superficial wall structure of MS/Ks, which can be seen in detail in Figure 4, seemed to be characterized by the overlap of several layers of polymer that were organized to form pentagonal, hexagonal, and octagonal structures. With regard to the processing conditions, for a lower PLGA content (MS/K-1 and MS/K-2), the particles were found to have a reticular structure with pore walls that were thicker than those observed with a higher PLGA content (MS/K-3 to MS/K-7). The increase in PVA seemed to not affect the particle morphology. With regard to the increase in the W2 volume, the MS/K-5 was found to be characterized by a reticular structure but with reduced open pores. The addition of PBS 0.01 M in W2 (hypoosmotic environment) was found to not influence MS morphology, while the addition of PBS 0.07 M (isosmotic environment) was found to lead to the formation of MSs with a smooth surface and small pores.

### 3.3. Diameter Distribution

**Figure 5:**
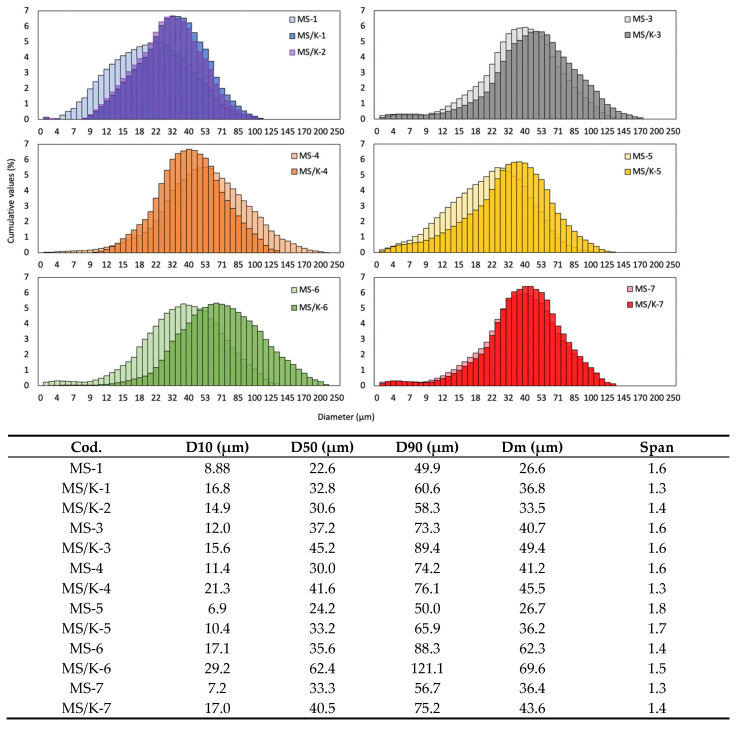
Effects of synthesis parameters on MS diameter distribution. The typical diameter distribution curve is reported in the exemplary graph. The values of the average diameter (D_M_—the diameters for which 90% (D90), 50% (D50), and 10% (D10) of the particles were below each value) and the span index are reported in the table.

The particle size distribution for all analyzed conditions is reported in Figure 5. As expected, both empty MSs and MS/Ks were found to have a mean diameter on the order of microns that ranged from 25 to 70 µm, which was in accordance with the production process [33].

With regard to the influence of the processing conditions on the empty MSs’ diameter distribution, the increase in PLGA concentration was directly related to the increase in the particles’ diameters for all conditions [32] except in the case of the MS-5, for which the increase in the W2 volume allowed us to reduce the particle diameter to values similar to those of MS-1 that were characterized by a lower PLGA content. In these two conditions, the high polydispersity index could have been due to the low PVA concentration for the MS-1 and to the high W2 volume for the MS-5 that tended to make the MS dispersion less homogeneous and stable. The size of the PLGA particles was reported to increase with the W2 volume. The formation of smaller particles with a higher volume ratio could have been due to a more efficient dispersion and stability of the primary emulsion in the W2 [32,50]. The increment in PVA content instead was found to not influence the particle diameter. With regard to the addition of PBS 0.01 and 0.07 M in the W2, it was responsible for a diameter increase in the case of PBS 0.01 M and for a decrease in the case of 0.07 M. In the case of the MS-6, the hyposmolarity of the W2 was responsible for the entry of the aqueous solution of W2 into the forming microspheres, which therefore assumed a larger diameter. On the contrary, in the case of the isosmolarity of W2 in MS-7, no variation in particle size was observed.

With regard to the influence of the processing conditions on the LYS-loaded particles, the same observations were made as in the case of the empty microspheres. The addition of LYS in the W1 was found to be responsible for the increase in the MSs’ diameter for all tested conditions that was visible as a shift in the cumulative value to higher values [32]. Moreover, the addition of L-lysine seemed to have a stabilizing effect because it reduced the span index value in the cases of MS/K-1, MS/K-2, MS/K-4, and MS/K-5 in addition to increasing their mean diameters. This effect was not visible in the MS/K-3, MS/K-6, and MS/K-7 conditions, for which a slight increase in the span index was registered.

### 3.4. Encapsulation Efficiency

The effects of the synthesis parameters on the EE% of the MS/Ks is reported in Table 4. The increase in the PLGA concentration led to a general increase in the EE% values, which was in accordance with the literature [32,36]. This was due to the fact that a higher polymer concentration allowed us to create MSs with a tighter mesh, which managed to more efficiently entrap a greater quantity of LYS. In other words, a higher polymeric solution viscosity could prevent the diffusion of the molecule of interest from the W1 to the W2 [33]. The W2 volume was increased with the aim of improving the EE% while relying on the evidence that a large W2 volume provides a faster diffusion of the organic solvent across the oil phase, thereby leading to a faster MS solidification [36]. As reported by Yeo et al., an increase in the W2 from 20 mL to 80 mL or more caused the MSs to quickly harden [36]. However, in MS/K-5, the increase in the W2 volume was responsible for a very low EE%. With regard to the PVA concentration, its increase directly influenced the EE%; that from MS/K-2 to MS/K-3 doubled and almost tripled in MS/K-4, which was in accordance with the literature [36]. With regard to the addition of PBS in the W2, the hyposmolarity of the MS/K-6 W2 caused a reduction in the EE%, while the isosmolarity of the MS/K-7 W2 did not cause an increase in the EE% value [26,32].

### 3.5. Release Kinetics

Normalized absorbance values were used to graph the LYS cumulative release profile of the MS/Ks over time; these were expressed as the percentage value of the total encapsulated amino acid for each sample or as the absolute weight of LYS released from each sample type. While the first graph provided information on the impact of processing on the MS/K release profile, the second allowed us to quantify and compare the effective amount of LYS released over time and to compare samples beyond their processing conditions.

**Figure 6:**
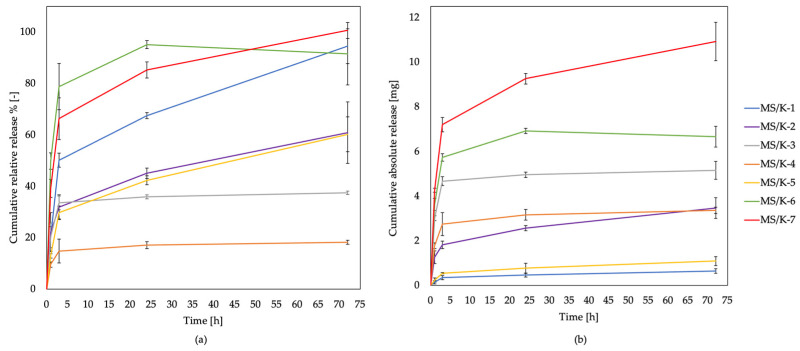
Cumulative relative (**a**) and absolute (**b**) release profiles of LYS from MS/K upon incubation in PBS at 37 °C for 72 h. LYS cumulative release was expressed as the released relative percentage of the total amount of encapsulated LYS or as the absolute weight of LYS released for each sample type. Reported values represent the mean ± SD (n = 6).

The MS/Ks showed an initial rapid release of LYS followed by a slow and continuous release (Figure 6). According to Makadia et al. and Ravi et al., the LYS release curve was found to be biphasic as a result of PLGA biodegradation [26,33]. The first phase was characterized by a drug burst release, a phenomenon that is related to the drug type, concentration, and polymer characteristics [26]. The drug located on the MS surface that was in contact with the release medium was released as a function of its solubility as well as of the water penetration rate into the polymer matrix [26,33]. In this step, the molecular weight of the polymer was progressively decreased by random scissions, but no significative weight loss and no soluble monomer product could be appreciated in this phase [26]. In second part of the curve, the polymer was hydrolyzed and the drug was released via diffusion and erosion until complete polymer solubilization occurred [26,33]. Thus, the LYS release profiles were all found to be characterized by a burst release within 3 h followed by a slow and constant release over time, which was in accordance with the literature on PLGA MSs produced via double emulsion [26,32,33]. The MS/K-1, MS/K-6, and MS/K-7 were found to almost completely release the encapsulated K within 72 h, which confirmed that a lower polymer content and a hypo- or isosmotic environment of W2 did not slow the drug release kinetics. The MS/K-7 had the highest release profile with a release of about 12 mg in 72 h; it was followed by MS/K-6 (about 6 mg) and MS/K-1 (about 1 mg). Moreover, the increase in the W2 volume negatively impacted not only the encapsulation efficiency but also the release kinetics. Accordingly, the MS/K-5 showed a release of about 50% of LYS in 72 h. Although its release profile seemed to be similar to that of the MS/K-2 shown in Figure 6a, it should be noted that their absolute release profiles were strongly different. Indeed, as shown in Figure 6b, the MS/K-5 was found to release less than 1 mg in 72 h, while MS/K-2 released about 3 mg in 72 h. The faster MS/K-2 release was ascribable to the lower PLGA and PVA contents compared to those of MS/K-3 and MS/K-4, while the faster MS/K-5 release rate was due to the W2-generated emulsion system instability. Thus, the increase in PLGA and PVA concentrations allowed us to obtain MSs with tighter meshes that in turn enabled us to strongly reduce the LYS release. Indeed, the LYS release was found to be limited to about 20–35% in the MS/K-3 and MS/K-4. In particular, the higher PLGA concentration allowed us to obtain a slower LYS release in the MS/K-3 compared to the other experimental conditions. Additionally, the increase in the PVA and PLGA concentrations in the MS/K-4 allowed us to obtain a slower LYS release rate compared to MS/K-3. A higher concentration of PVA in the W2 led not only to an increase in the EE% but also to a decrease in the initial burst [36]. Thus, the MS/K-4 was found to allow a slow LYS release of about 20% (about 3 mg) in 72 h. The MS/K-3, which showed a 37% release, discharged about 5 mg of LYS within 72 h. Therefore, the obtained results confirmed that by modifying some MS/K production process parameters, it was possible to customize the DDS release profile according to the specific needs.

### 3.6. Degradation Resistance

**Figure 7:**
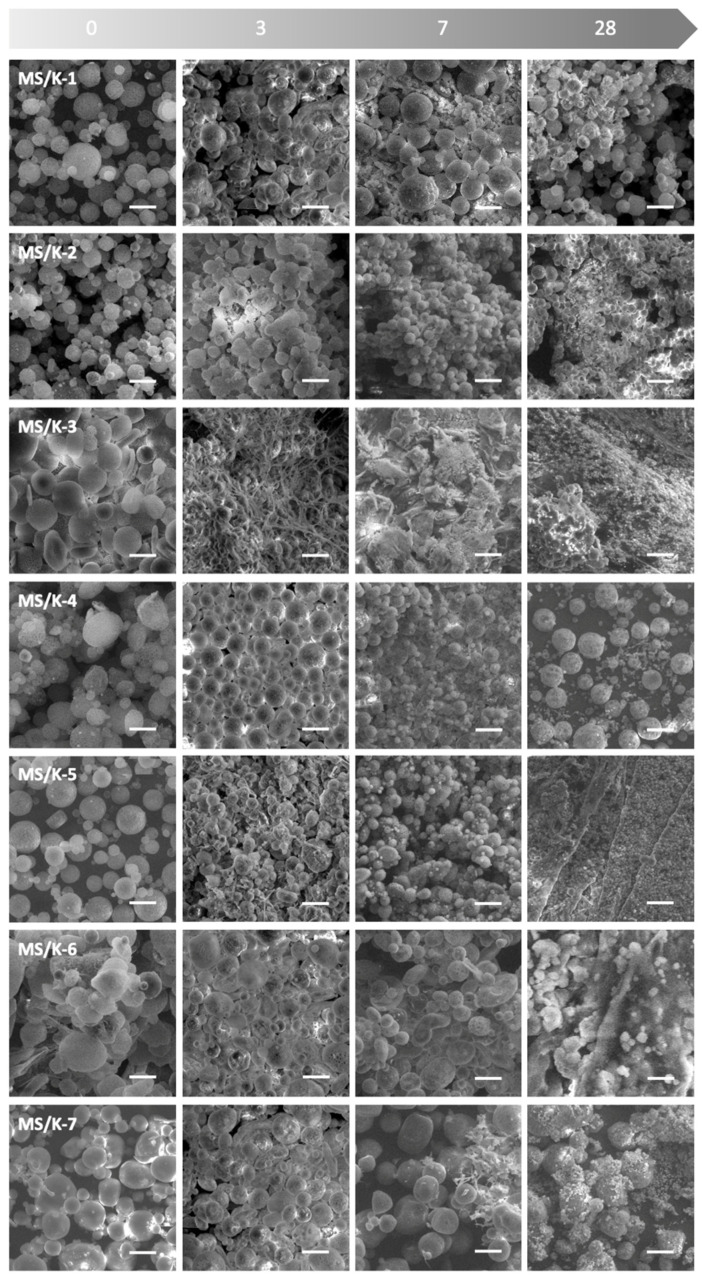
Degradation resistance of MS/Ks in a physiological-like environment (PBS 1X, pH 7.4, 37 °C) up to 28 days as observed via SEM. Scale bar: 50 µm; magnification: 500×.

The degradation resistance of the MS/Ks was qualitatively observed via SEM. PLGA is a typical bulk-eroding biopolymer and, as is well known, drug release from PLGA (50:50) MSs occurs in two phases [26,51,52]. The first phase was characterized by a rapid decrease in the PLGA molecular weight with a negligible mass loss, whereas the second phase was characterized by a slow reduction in the PLGA molecular weight but a fast weight loss [51]. As reported in Figure 7, the MS/K integrity was almost unchanged on day 3 and begun to be visible after 7 days. The synthesis parameters were found to influence the MS/K degradation. In particular, the MS/K-1 and MS/K-2 showed a similar degradation profile with degradation signs from the 7th day, which was ascribable to the lower PLGA and PVA concentrations compared to the other samples. Despite this, the particles were still clearly visible and distinguishable after 28 days. The increase in the PLGA and PVA concentrations in the MS/K-3 was found to be responsible for an early degradation that started after 3 days and became even more evident after 28 days when the microparticles were still visible but partially fused together and thus not well distinguishable, which probably was ascribable to the non-optimal processing conditions (i.e., an insufficient amount of surfactant for a high polymer concentration). The further increase in PVA in the MS/K-4 instead resulted in higher MS stabilization. In particular, degradation aggregates could be observed after 7 days of incubation, and after 28 days the particles were found to be almost completely intact. The emulsion system’s instability in the MS/K-5 caused by a 10× increase in the W2 volume resulted in their fast degradation. Indeed, MS/K-5 degradation began after 3 days, and after 28 days the particles were almost degraded because they were still visible but were partially fused together and not well distinguishable. With regard to the synthesis in a hypo- or isosmotic W2 environment, we found that the presence of PBS 0.01 M in the W2 caused the MS/K-6 to degrade faster (the degradation was almost complete after 28 days), while the presence of PBS 0.07 M in W2 did not show a degradation behavior similar to that of MS/K-1.

### 3.7. In Vitro Response

#### 3.7.1. MS/K-4 Biocompatibility Assessments with HMEC-1

Because wound healing may require a slow and constant release system of LYS to properly sustain angiogenesis throughout the period required for regeneration of injured tissue, the MS/K-4 was selected for its highest EE% content and lowest release kinetics. Accordingly, the in vitro response of human microvascular endothelial cells (HMEC-1) to the MS/K-4 was studied to evaluate the biocompatibility and its potential as a system for the controlled release of LYS to enhance angiogenesis.

**Figure 8:**
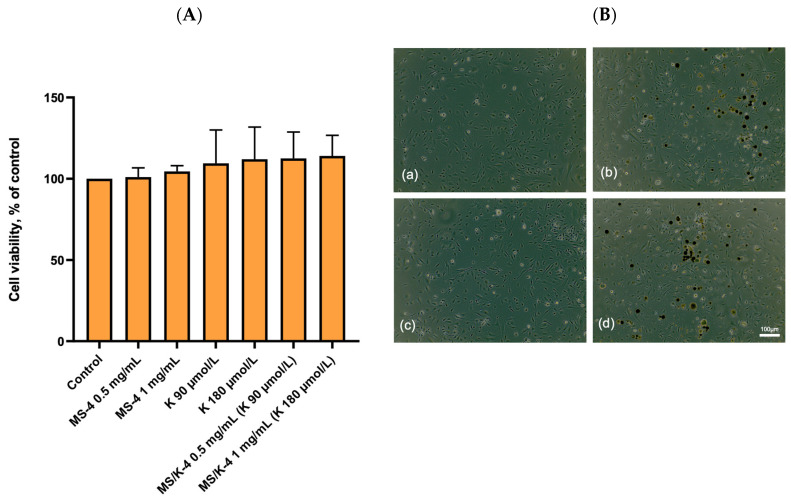
Effects of MS/K-4 on cell viability. HMEC at confluence was treated with MS/K-4, LYS, or MS-4 for 24 h at the concentrations indicated. (**A**) Cell viability was assessed via an MTT assay and was expressed as the percent of the basal (untreated) control. Data (means ± S.D.; n = 3) were expressed as a percent of the untreated control. (**B**) Images of the cell monolayer visualized and acquired with a phase-contrast microscope at 10× magnification: (**a**) control; (**b**) MS-4 1 mg/mL; (**c**) LYS 180 µmol/L; (**d**) MS/K-4 1 mg/mL.

**Figure 9:**
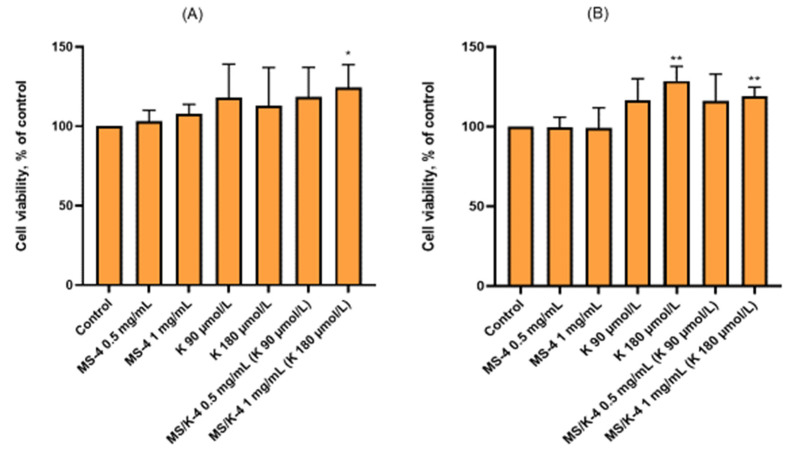
Effects of MS/K-4 on cell viability. (**A**) HMEC was seeded at low confluence (10,000 cells/cm^2^) and treated after 6 h with MS/K-4, free LYS, or MS-4 for 24 h at the concentrations indicated. Cell viability was then assessed via an MTT assay and was expressed as the percent of the basal (untreated) control. Data (means ± S.D.; n = 3) were expressed as the percent of the untreated control. (**B**) HMEC was seeded at low confluence (10,000 cells/cm^2^) in the presence of MS/K-4, LYS, or MS-4 for 24 h at the concentrations indicated. Cell viability was then assessed via an MTT assay and was expressed as the percent of the basal (untreated) control. Data (means ± S.D.; n = 3) were expressed as the percent of the untreated control. * *p* < 0.05 vs. basal (untreated) control; ** *p* < 0.01 vs. basal (untreated) control.

To evaluate the safety of empty PLGA microspheres and LYS-loaded PLGA microspheres, the cytotoxicity of both formulations was assessed on HMEC-1 under quiescent, non-proliferating conditions (Figure 8) and under proliferating conditions (Figure 9A,B) via an MTT assay.

As shown in Figure 8, the addition of the MS/K-4 or MS-4 to confluent endothelial cells for 24 h had no effect on the cell viability and/or metabolic activity, which indicated the complete biocompatibility of the proposed formulations.

Comparable results were obtained in the presence of free LYS, which was tested at the higher concentrations that could be achieved after prolonged release of the MS/K-4. Accordingly, the treated cells showed no change in the typical cobblestone morphology of the vascular endothelium when examined morphologically under a phase-contrast microscope (Figure 8B, right panels).

However, under conditions that promote the activation of angiogenic processes, endothelial cells are stimulated to proliferate and undergo profound phenotypic and metabolic changes. For this reason, we decided to test the MS/K-4 on adherent but still proliferating cells. As shown in Figure 9A, both the MS-4 and MS/K-4 and the LYS had no toxic effects, but the MS/K-4 significantly increased the MTT reduction at higher concentrations as an index of the increased cellular metabolic activity and/or viability. Even more interesting were the results observed when the cells were seeded in the presence of the MS/K-4 (Figure 9B). Even under these tricky metabolic conditions, we did not observe any impairment in the adhesion of endothelial cells to the gelatin-coated flask surface by the MS/K-4. Nevertheless, at higher concentrations the MS/K-4 significantly increased the cellular metabolic activity and/or viability of the treated endothelium.

#### 3.7.2. MS/K-4 Stimulated Angiogenesis: Effects on Cell Migration

**Figure 10:**
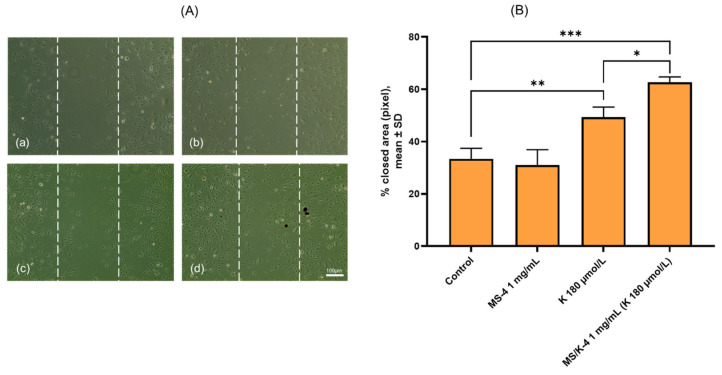
MS/K-4 induced angiogenesis in human endothelial cells. HMEC-1 was treated with MS/K-4, free LYS, or MS-4 for 24 h at the concentrations indicated before vertical scratching of the monolayer. After 24 h, cell migration of (**a**) control, (**b**) MS-4 1 mg/mL, (**c**) LYS 180 μmol/L, (**d**) MS/K-4 1 mg/ml was observed using phase-contrast microscopy (**A**) and quantified using ImageJ software optimized with specific plugins to automatically detect the scratched area (**B**). Data (mean ± S.D.; n = 3) were expressed as the closed wound area. * *p* < 0.05 vs. basal (untreated) control; ** *p* < 0.01 vs. basal (untreated) control; *** *p* < 0.001 vs. basal (untreated) control.

A crucial event in orchestrating angiogenesis and ensuring vessel growth is the directional migration of endothelial cells toward denuded, cell-empty areas. For this reason, we first tested the pro-angiogenic potential of the MS/K-4 by performing a functional angiogenesis assay called the scratch assay, which measured the acquisition of motility properties by the endothelial cells when contact inhibition was lost and cells were subsequently stimulated to migrate [53]. As shown in Figure 10, 24 h exposure of endothelial cells to the MS/K-4 in conjunction with cell detachment significantly increased the migration rate of HMEC-1 toward the acellular wound area compared with the migration rates of the same cells under control (untreated) conditions and even compared with maximal LYS concentration (Figure 10). Indeed, compared with the untreated control condition, LYS resulted in a 45% increase in the area covered by the cells, whereas the MS/K-4 resulted in a 60% increase in wound closure. Cell exposure to LYS only (always tested at the higher concentrations that could be reached after prolonged release of the MS/K-4) resulted in a significant increase in the cell mobility (*p* < 0.01 compared to the untreated control condition) but did not reach the efficacy of the MS/K-4 (*p* < 0.01; LYS compared to MS/K-4).

#### 3.7.3. MS/K-4 Stimulated the Expression of Pro-Angiogenic Genes in the Endothelium

To gain insight into the mechanism by which the MS/K-4 could support and/or promote angiogenesis, we also preliminarily examined the proangiogenic effect of the MS/K-4 by analyzing the mRNA expression of several key proangiogenic genes. These included metalloproteinase (MMP)-9, which is involved in the degradation of the extracellular matrix, thereby allowing cell movement [54]; and focal adhesion kinase (FAK), which in turn is involved in regulating cytoskeletal reorganization, cell adhesion, growth, survival, and migration [55]. In addition, we also studied the expression of several important proangiogenic factors that included vascular endothelial growth factor (VEGF) and its related receptor KDR/flk-1; platelet-derived growth factor (PDGF); fibroblast growth factor (FGF)-2 [56]; and the expression of endothelial nitric oxide synthase (eNOS), the activity of which supports the angiogenic activity of several growth factors, including that of VEGF [57].

**Figure 11:**
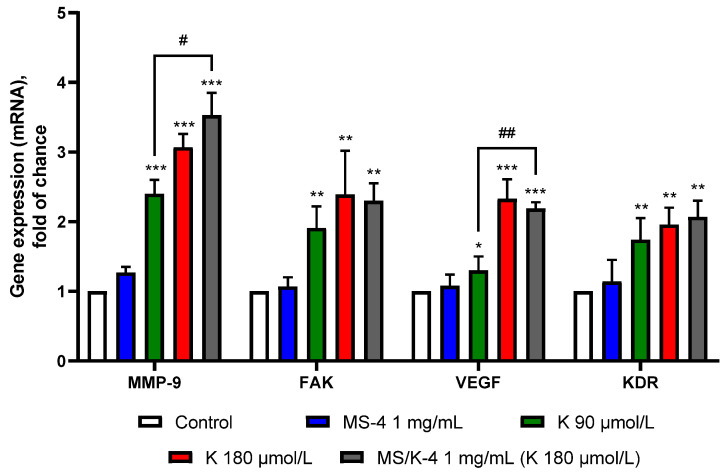
MS/K-4 induced the expression of pro-angiogenic genes in human endothelial cells. HMEC-1 was treated with MS/K4, free LYS, or MS-4 for 24 h at the concentrations indicated. The total RNA was then extracted from cells, and the mRNA levels of MMP-9, FAK, VEGF, and KDR were measured via qPCR using specific primers and normalized to GAPDH RNA. Data (means ± S.D.; n = 3) were expressed as fold induction over the basal (untreated) control. * *p* < 0.05 versus basal (untreated) control; ** *p* < 0.01 vs. basal (untreated) control; *** *p* < 0.001 vs. basal (untreated) control; # *p* < 0.05 between joined bars; ## *p* < 0.01 between joined bars.

First, we examined the MS/K-4 activity on mRNA expression of two genes characteristic of cell mobility and proliferation: MMP-9 and FAK. As shown in Figure 11, the expression of the mRNAs of MMP-9 and FAK was significantly induced by the MS/K-4 (*p* < 0.001 versus the untreated control). The proangiogenic role of the encapsulated LYS was confirmed when cells were exposed to free LYS at high (180 µmol/L) and intermediate (90 µmol/L) concentrations (*p* < 0.001 versus the untreated control), which also induced the expression of both genes. Interestingly, despite the low release of LYS from the encapsulated formulation (≈30 µmol/L after 24 h), we observed a significantly higher activity for the MS/K-4 in terms of MMP-9 and VEGF gene induction (*p* < 0.001 between the MS/K-4 compared with LYS at 90 µmol/L).

Since FAK activity has been shown to induce *KDR/flk-1* gene expression in adult endothelial cells [58], we tested the effects of the MS/K-4 on the expression of KDR and VEGF mRNAs. Consistent with the FAK mRNA increase, the expression of KDR and VEGF mRNAs was increased by cell exposure to the MS/K-4, which provided further confirmation of an angiogenesis-stimulating role of LYS.

**Figure 12:**
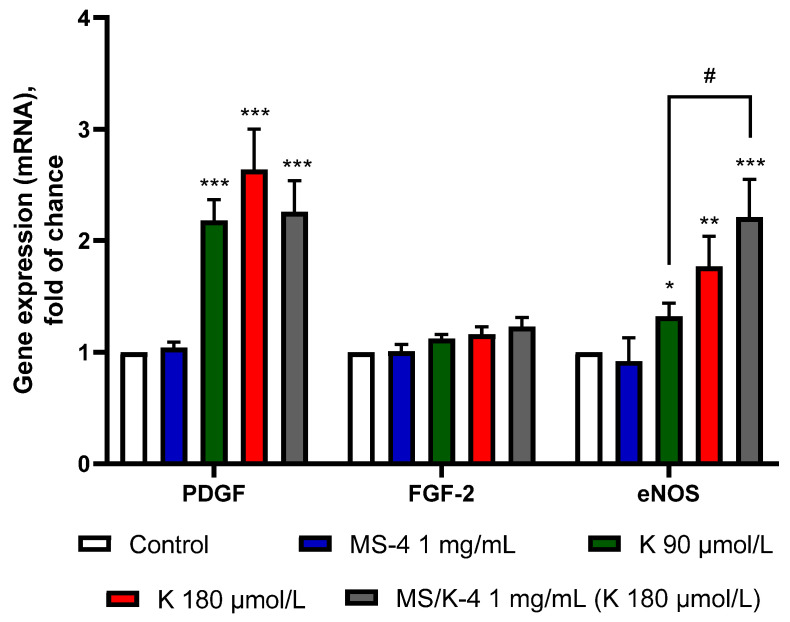
MS/K-4 induced the expression of pro-angiogenic genes in human endothelial cells. HMEC-1 was treated with MS/K-4, free LYS, or MS-4 for 24 h at the concentrations indicated. The total RNA was then extracted from cells, and the mRNA levels of PDGF and FGF-2 were measured via qPCR using specific primers and normalized to GAPDH RNA. Data (means ± S.D.; n = 3) were expressed as fold induction over the basal (untreated) control. * *p* < 0.05 versus basal (untreated control); ** *p* < 0.01 vs. basal (untreated control); *** *p* < 0.001 vs. basal (untreated) control; # *p* < 0.05 between joined bars.

Finally, we examined the effects of the MS/K-4 on the mRNA expression of PDGF and FGF-2, two growth factors that are known for their characteristic synergistic activity in stimulating angiogenic vascular growth [59] and in the gene expression of eNOS. As shown in Figure 12, the MS/K-4 was able to increase the expression of PDGF and eNOS, whereas the expression of FGF was not affected. Despite the lower release of LYS from the encapsulated formulation, we observed significantly higher activity for the MS/K-4 in terms of eNOS gene induction (*p* < 0.001 between the MS/K-4 compared with LYS at 90 µmol/L), which indicated an enhancement of the LYS activity under controlled release conditions.

## 4. Discussion

Vascularization plays a key role in tissue homeostasis, repair, and healing. However, in cases of injuries or diseases, this complex and finely regulated process is not sufficient to restore the vascular perfusion and thus recover the tissue’s physiological function. The field of tissue engineering has made remarkable progress in developing strategies to restore, ameliorate, or replace the function of damaged tissues within the body. However, the success of engineered tissues has been limited by the absence of the adequate vascularization required to sustain tissue growth/remodeling [1]. Notwithstanding, tissue engineering has attempted to develop several types of biomaterial-based strategies to enhance vasculogenesis by providing pro-angiogenic agents (i.e., growth factors, cells, cell-derived materials, decellularized matrices, antibodies, peptides, DNA, and RNA) in a controlled manner (i.e., through ad hoc DDSs).

In this work, with the ultimate purpose to develop advanced therapies for the treatment of hard-to-heal wounds, a drug-loaded, microparticulate-based delivery system was developed in order to support angiogenesis and regenerative processes. In particular, the system was developed to supply LYS, an essential amino acid with pro-angiogenic and pro-healing activity [16,17,18,19,20,21], to the damaged tissue in a controlled and constant manner over time. Among several existing biomaterials for the development of DDSs, we chose PLGA, an FDA-approved synthetic polymer due to its high versatility, biocompatibility, biodegradability, and tunable properties as an encapsulating material. The choice of a manufacturing material with biodegradation products that were able to sustain pro-regenerative processes [5,29,30] allowed us to ensure the controlled trigger of cell response in the long term. Thus, LYS-loaded PLGA MSs were designed to stimulate angiogenesis and wound-healing processes by maintaining the controlled release of LYS in the short term and the slow release of PLGA degradation products in the long term. To the best of our knowledge, this was the first study that dealt with the development, optimization, and (preliminarily) the bioactivity evaluation of a microparticulate system for the controlled release of LYS. In order to optimize and customize the LYS encapsulation efficiency and release kinetics, the impacts of some processing parameters on the MS properties were investigated.

First of all, the morphological analysis confirmed the effective production of PLGA MSs of about 20–40 μm [32,33]. As reported in the literature, the optimal drug delivery was achieved by using MSs with diameters in the range of 10–200 μm [60]: MSs with diameters <10 μm would be phagocytosed by immune cells [61]; while on the other hand, MSs with diameters >200 μm may cause immune response and inflammation [61]. A smooth surface with small pores was observed for all empty formulations, which was in accordance with the literature [32]. On the contrary, LYS loading was found to deeply affect the particles’ morphology, which showed the acquisition of a reticular structure.

We found that the addition of LYS was responsible not only for the change in the morphology of the MSs but also for the increase in the particle size distribution, which was reflected in a shift in the cumulative diameter distribution to higher values [32]. The particle size distribution also was revealed to be influenced by the processing conditions. In particular, the MS diameter distribution was revealed to be directly proportional to the PLGA concentration [32] and indirectly proportional to the W2 volume [50].

An evaluation of the MS yield allowed us to confirm that the described process enabled us to recover up to about 70–80% of the microspheres. This result indicated that the double-emulsion technique, the chosen solvents, and the key process parameters (i.e., the temperature, ratio between the individual phase volumes, homogenization speed, and time) allowed us to transform almost all of the raw polymer into microparticles.

The synthesis parameters were found to deeply affect the EE%. In particular, the EE% was found to be directly proportional to the PLGA and PVA concentrations [32,33,36] and inversely proportional to that of the W2 [36]. The hyposmolarity of the W2 caused the reduction in the EE%, while the isosmolarity of the W2 did not cause the EE% increase [32]. The higher EE% of about 36% was reached by the MS/K-4 by increasing both the PLGA and PVA concentrations.

With regard to the LYS release kinetics, as expected, all MS/K specimens showed an initial rapid release of LYS within 3 h followed by a slow and continuous release [26,32,33].

Moreover, it was clear that by modifying some of the MS/K production process parameters, it was possible to customize the LYS delivery system’s release profile according to specific needs. In particular, we observed that a lower polymer content and a hypo- or isosmotic environment of the W2 did not slow the drug release kinetics because an almost complete release of the encapsulated LYS was reached within 72 h. The increases in the PLGA and PVA concentrations allowed us to strongly reduce the LYS release, which was found to be limited to about 20–35% of the total encapsulated LYS. Among all formulations, the MS/K-4 was found to allow the slowest LYS release of about 20% in 72 h, while MS/K-7 was found to be the fastest.

The MS/K-4 was selected for a subsequent biological evaluation because the long-term controlled and prolonged release of bioactive factors is essential for in vivo efficacy. The MS degradation was qualitatively observed via SEM. As reported in Figure 7, the MS/K integrity was almost unchanged on day 3 and began to be visible after 7 days and became even more so by day 28. The increase in the PLGA and PVA concentrations gave the MSs a higher stabilization. The increase in the W2 volume resulted in a fast degradation of the MSs. The presence of PBS 0.01 M in the W2 caused the MSs to degrade faster (the degradation was almost complete after 28 days), while the presence of PBS 0.07 M in the W2 did not.

The optimized formulation (MS/K-4) was biologically characterized in vitro for its biocompatibility and stimulation of angiogenesis. In agreement with several previous reports [62], a promising biocompatibility of the PLGA-based MSs with endothelial cells was observed. The PLGA-based MSs were indeed free of toxic effects and did not affect the cell survival under quiescent conditions that simulated the stable, non-proliferating phenotype of adult endothelial cells under the condition of contact inhibition [63]. Of note, the addition of MS/K-4 stimulated cell metabolism or cell population growth, which suggested that the targeted delivery of LYS may support the stimulus for proliferation. To further support the complete biocompatibility of the MS/K-4 and the potential stimulation of cell proliferation, we also observed that the addition of the MS/K-4 during cell seeding did not affect the adhesion properties of endothelial cells and even supported a significant increase in the cell metabolism/proliferation similar to that for high LYS concentrations.

The pro-angiogenic potential of the MS/K-4 in cell migration—an essential condition that underlies angiogenesis [53]—was then tested. Consistent with our hypothesis, whereas MS-4 did not promote cell mobility, LYS and especially the MS/K-4 induced cell mobility; the effect was significantly higher in LYS-loaded MSs, which suggested a deep influence of LYS on the cell metabolism associated with locomotion [64] as well as an improved wound healing by the lysine-loaded particles. Intriguingly, the ameliorative effect of the encapsulated LYS compared to the free LYS was probably due to the controlled release, which avoided the default partial degradation/inactivation (of free LYS) by the complex of redox/neutralization/sequestration agents that comprised the complete growth medium. This aspect should further enhance the interest in our results as prototypically applicable to other compounds. Because cell migration is triggered by mechanotactic stimuli and also involves the degradation of the extracellular matrix to allow the progression of migrating cells [53], the effect of the MS/K-4 was on the expression of a number of genes involved in cell signaling that was triggered when cells were free to move; these genes included *FAK* and *MMP-9*.

In addition, at the level of mRNA expression, the LYS-loaded MSs exerted a triggering effect and induced increases in FAK, MMP-9, and eNOS mRNA as well as in mRNAs of proangiogenic genes that are closely related to FAK activity (such as KDR and its ligand VEGF).

Overall, our data demonstrated the biocompatibility of the MS/K-4 and a clear supportive effect in activating angiogenesis pathways in vitro. They also provided detailed hints toward understanding how LYS can contribute to these effects, which should be of a particular interest in understanding the angiogenesis process and further implementing proangiogenic strategies.

Our results, which suggested the best efficacy of lysine-loaded particles in supporting functional angiogenesis, warrant the usefulness of further experimental studies. The sustained release of lysine by the MS/K-4 may have partially contributed to the greater efficacy of MS/K-4 over the free lysine in promoting wound healing. However, further studies are needed to deepen the understanding of the uptake of charged lysine particles by endothelial cells, the release of lysine, and the role in the angiogenic process. In addition, our study was performed in a preclinical phase using human endothelial cell cultures, a model that is known to be a reliable and versatile tool for studying in vitro angiogenesis in its basic activation before facing the complexity of the in vivo context. Nevertheless, further studies (including human studies) are needed to confirm and further evaluate the in vivo efficacy of loaded lysine particles in promoting angiogenesis and wound repair.

## 5. Conclusions

The modulation of neovascularization represents a potential therapeutic tool against a large number of diseases. With regard to this, in this work a novel PLGA-based system for the delivery of the pro-angiogenic amino acid LYS was developed, characterized, and proposed as new, simple, and effective tool for angiogenesis enhancement in injured tissues. All analyses confirmed that the chosen production process, solvents, and key process parameters (i.e., the temperature, ratio between the individual phase volumes, homogenization speed, and time) allowed to transform almost all of the raw polymer into MS/K specimens of about 20–40 μm with tunable LYS release profiles. Cell studies proved the MS/Ks’ pro-angiogenic power, thereby paving the way toward the development of new advanced therapies and strategies for the treatment of hard-to-heal injured tissues. Based on these findings, further studies should be performed to reach an in-depth understanding of the pro-angiogenic activity of MS/Ks.

## Figures and Tables

**Table 1:** Investigated processing parameters of MSs.

Cod.	W1		O		W2	
	LYS (%)	V_W1_ (mL)	V_O_ (mL)	PLGA (%)	V_W2_ (mL)	PVA (%)
MS-1	2	0.5	5	10	50	0.5
MS-2	10	0.5	5	10	50	0.5
MS-3	10	0.5	5	15	50	1
MS-4	10	0.5	5	15	50	2
MS-5	10	0.5	5	15	500	1
MS-6	10	0.5	5	15	50	1, PBS 0.01 M
MS-7	10	0.5	5	15	50	1, PBS 0.07 M

**Table 2:** Primer sequences used for qPCR analysis.

Gene Symbol	Full Name	Forward Primer (5′-3′)	Revers Primer (3′-5′)	Accession Number
VEGF	Vascular endothelial growth factor	GACACACCCACCCACATACA	TCTCCTCCTCTTCCCTGTCA	NM_001171626.1
KDR/VEGFR-2	Kinase insert domain receptor/vascular endothelial growth factor receptor 2	AGCGATGGCCTCTTCTGTAA	ACACGACTCCATGTTGGTCA	NM_002253.2
MMP-9	Matrix metalloproteinase-9	TTGACAGCGACAAGAAGTGG	GCCATTCACGTCGTCCTTAT	NM_004994.2
FAK	Focal adhesion kinase	ATTAAATGGATGGCTCCA	CTCCCACATACACACACC	MN_001352694.2
PDGF	Platelet-derived growth factor	TTGTGCGGAAGAAGCCAATC	CTCCTTCAGTGCCGTCTTGT	NM_033016.3
FGF-2	Basic fibroblast growth factor-2	AGAGCGACCCTCACATCAAG	TCGTTTCAGTGCCACATACC	NM_002006.5
eNOS	Endothelial nitric oxide synthase	ACCCTCACCGCTACAACATC	GCTCATTCTCCAGGTGCTTC	NM_000603.4
GAPDH	Glyceraldehyde-3-phosphate dehydrogenase	ATGGCCTTCCGTGTCCCCAC	ACGCCTGCTTCACCACCTTC	NM_002046.3

**Table 3:** Effects of the variations in synthesis conditions on MS and MS/K yields. Reported values represent the mean ± SD (n = 3).

Cod.	Yield (%)
MS-1	71.8 ± 1.7
MS/K-1	70.6 ± 1.2
MS/K-2	74.2 ± 0.9
MS-3	69.3 ± 1.8
MS/K-3	90.1 ± 0.6
MS-4	98.4 ± 1.1
MS/K-4	88.3 ± 1.3
MS-5	80.6 ± 0.8
MS/K-5	84.0 ± 1.0
MS-6	78.6 ± 0.7
MS/K-6	84.0 ± 0.9
MS-7	85.3 ± 1.5
MS/K-7	86.5 ± 1.4

**Table 4:** Effects of synthesis parameters on MS/K encapsulation efficiency (EE%). Reported values represent mean ± SD (n = 3).

Sample	LYS_i_ (mg)	LYS_f_ (mg)	EE (%)
MS/K-1	10	0.9 ± 0.3	8.9 ± 1.8
MS/K-2	50	6.2 ± 1.0	12.4 ± 2.0
MS/K-3	50	13.2 ± 1.4	25.9 ± 3.2
MS/K-4	50	18.0 ± 1.6	36.0 ± 2.3
MS/K-5	50	1.8 ± 0.8	3.6 ± 2.2
MS/K-6	50	8.2 ± 1.4	16.4 ± 3.4
MS/K-7	50	11.9 ± 2.1	23.7 ± 2.0

## Data Availability

Not applicable.
